# Supplementary material for: HTSlib: C library for reading/writing high-throughput sequencing data
Source: Gigascience. 2021 Feb 16;10(2):giab007. doi: 10.1093/gigascience/giab007 (PMC7931820; doi:10.1093/gigascience/giab007)
Supplement: giab007_Response_to_Reviewer_Comments_Original_Submission [file giab007_response_to_reviewer_comments_original_submission.pdf]

Author's Response To Reviewer Comments

Close

Submission ID: GIGA-D-20-00368
Title: HTSlib - C library for reading/writing high-throughput sequencing data

Response to Reviewers

We would like to thank the editor and reviewers for careful review of our manuscript. Their suggestions and
comments were helpful for us to refine this paper. Please see the list of revisions and our responses highlighted below.

Sincerely,
Andrew Whitwham et al.

Reviewer #1: Although not directly involved in this work, I have witnessed major events leading to current C library of HTSlib. As indicated by the authors, there was a strong demand in the genomic community to unify various short-read alignment formats and to code genome variation. As the accumulation of genome sequencing data is still accelerating, an efficient solution in both space and time is required. Currently this library is maintained and further developed by experts at the front and various new features are being introduced to meet new demands. I am glad that this library is freely available for commercial and non-commercial use, which is vital for the field. Here are a few minor suggestions.
1) Starting from VCF format, small variation is essentially the major category among many to drive the improvement. There were attempts to code structural variants as multiple breakpoints, contradicting current one line one variant practice. More complex structural variation will emerge when pacbio HiFi is applied, especially in cancer studies. Although this issue is not yet possible to solve right now, the procedure about how HTSlib team interacts with broader genomics community to discuss and absorb ideas could be described.

Thank you for the suggestion. The Discussion section briefly mentions the expected structural variation changes in VCF. Since there is an overlap between VCF specification maintainers and HTSlib maintainers, and GA4GH is an implementation-lead organisation we expect to be implementing this once the specification becomes more concrete. To make this clearer, we updated the relevant part of the Discussion to read:
"Over the years various improvements and modifications have been made to the specifications. Together these have been and will continue to be a driving force for continued development."

2) In certain performance tests, a RAM disk is used. Although this does provide theoretical throughput and mimics data flow from a pipe, it might not be what regular users would experience in their daily data processing. Thus, perhaps all the tests could be unified with SSD as the storage device.

Thank you for the suggestion, this is a good suggestion. We did consider adding such tests, however, because I/O limits of the hardware are highly variable with many different possible configurations, we choose to present the theoretical best performance. In order to aid spotting the likely I/O bottlenecks we also now report figures in megabytes per second, with colouring for typical HDD and SDD bandwidth limits.

3) It is wonderful that HTSlib includes remote data transfer protocols and I personally consider it particularly powerful once network speed enables stream computing. However, current speed tests are for local data only. It will be great if performance of remote data transfer protocols could be demonstrated in the supplementary material.

Similarly to the previous point, the performance over network is also highly variable and very hard to measure in a robust way, therefore we choose not to include such tests. Network performance is typically dominated by a mix of total data volume and the number of seeks, which will become separate network round-trips. These numbers are reported in tables S6 and S7.

Reviewer #2: Solid summary article for a great piece of infrastructure. Just a few nits:

Background: The reported timing of HTSlib's creation is inconsistent: the end of the second paragraph refers to the 2013 addition of CRAM to HTSlib (and later, the first Discussion sentence, regarding 100-fold reduction in sequencing costs, refers to a 2008-09 starting point), but the fourth paragraph refers to the library's creation in 2014. The text should be edited to more clearly distinguish the proto-HTSlib component of SAMtools from the dedicated 2014- library.

Thank you for pointing out these inconsistencies. We updated the text to clarify these apparent discrepancies, clarifying both the project start and first official release dates.

Figures 2, 4, and S9 are bar charts with log-scale y-axes; this is not a great combination, since bar charts encourage comparison of bar areas.

Thank you for the suggestion. We updated the graphs as log-scale y-axis is not really necessary.

Close
