# Supplementary material for: HTSlib: C library for reading/writing high-throughput sequencing data
Source: Gigascience. 2021 Feb 16;10(2):giab007. doi: 10.1093/gigascience/giab007 (PMC7931820; doi:10.1093/gigascience/giab007)
Supplement: giab007_Reviewer_1_Report_Original_Submission — Kai Ye -- 12/31/2020 Reviewed [file giab007_reviewer_1_report_original_submission.pdf]

**Reviewer Report**

**Title: HTSlib - C library for reading/writing high-throughput sequencing data**

**Version: Original Submission** **Date:** 12/31/2020

**Reviewer name: Kai Ye**

**Reviewer Comments to Author:**

Although not directly involved in this work, I have witnessed major events leading to current C library of HTSlib. As indicated by the authors, there was a strong demand in the genomic community to unify various short-read alignment formats and to code genome variation. As the accumulation of genome sequencing data is still accelerating, an efficient solution in both space and time is required. Currently this library is maintained and further developed by experts at the front and various new features are being introduced to meet new demands. I am glad that this library is freely available for commercial and non-commercial use, which is vital for the field. Here are a few minor suggestions.

1) Starting from VCF format, small variation is essentially the major category among many to drive the improvement. There were attempts to code structural variants as multiple breakpoints, contradicting current one line one variant practice. More complex structural variation will emerge when pacbio HiFi is applied, especially in cancer studies. Although this issue is not yet possible to solve right now, the procedure about how HTSlib team interacts with broader genomics community to discuss and absorb ideas could be described.
2) In certain performance tests, a RAM disk is used. Although this does provide theoretical throughput and mimics data flow from a pipe, it might not be what regular users would experience in their daily data processing. Thus, perhaps all the tests could be unified with SSD as the storage device.
3) It is wonderful that HTSlib includes remote data transfer protocols and I personally consider it particularly powerful once network speed enables stream computing. However, current speed tests are for local data only. It will be great if performance of remote data transfer protocols could be demonstrated in the supplementary material.

**Level of Interest**

Please indicate how interesting you found the manuscript: Choose an item.

**Quality of Written English**

Please indicate the quality of language in the manuscript: Choose an item.

**Declaration of Competing Interests**

Please complete a declaration of competing interests, considering the following questions:

- Have you in the past five years received reimbursements, fees, funding, or salary from an organisation that may in any way gain or lose financially from the publication of this manuscript, either now or in the future?
- Do you hold any stocks or shares in an organisation that may in any way gain or lose financially from the publication of this manuscript, either now or in the future?
- Do you hold or are you currently applying for any patents relating to the content of the manuscript?
- Have you received reimbursements, fees, funding, or salary from an organization that holds or has applied for patents relating to the content of the manuscript?
- Do you have any other financial competing interests?
- Do you have any non-financial competing interests in relation to this paper?

If you can answer no to all of the above, write 'I declare that I have no competing interests' below. If your reply is yes to any, please give details below.

I declare that I have no competing interests

I agree to the open peer review policy of the journal. I understand that my name will be included on my report to the authors and, if the manuscript is accepted for publication, my named report including any attachments I upload will be posted on the website along with the authors' responses. I agree for my report to be made available under an Open Access Creative Commons CC-BY license (http://creativecommons.org/licenses/by/4.0/). I understand that any comments which I do not wish to be included in my named report can be included as confidential comments to the editors, which will not be published.

Choose an item.

To further support our reviewers, we have joined with Publons, where you can gain additional credit to further highlight your hard work (see: https://publons.com/journal/530/gigascience). On publication of this paper, your review will be automatically added to Publons, you can then choose whether or not to claim your Publons credit. I understand this statement.

Yes Choose an item.
